# Preservation Study for Raw Conditioned Mutton During Refrigerated Storage by Food Preservatives

**DOI:** 10.3390/foods14091579

**Published:** 2025-04-30

**Authors:** Jiying Qiu, Junhua Wang, Shuangzhi Zhao, Yang Li, Jinyu Yang, Xingwang Zhang, Liang Wang, Xiaoxiao Jiang, Xiangyan Chen, Leilei Chen, Qingxin Zhou

**Affiliations:** 1 Institute of Food & Nutrition Science and Technology, Shandong Academy of Agricultural Sciences, Key Laboratory of Novel Food Resources Processing, Ministry of Agriculture and Rural Affairs, Jinan 250100, China; qjyfood@163.com (J.Q.); wangjunhua85@126.com (J.W.); zhaoshz717@163.com (S.Z.); liyang8654@163.com (Y.L.); yangjinyu86@163.com (J.Y.); 13969108617@163.com (X.Z.); 18369907775@163.com (L.W.); jxx5186@163.com (X.J.); chenxy@saas.ac.cn (X.C.); zhouqx0211@163.com (Q.Z.); 2 College of Animal Science and Technology, Shandong Agricultural University, Tai’an 271018, China; 3 College of Life Science, Shandong Normal University, Jinan 250014, China

**Keywords:** meat preparation, bacterial community diversity, spoilage bacteria, *Serratia liquefaciens*, ε-polylysine hydrochloride, sodium diacetate

## Abstract

The quality modification of chilled, raw conditioned mutton (RCM) after storage significantly impacts consumer preferences, making shelf-life extension and quality preservation crucial. This study evaluated the effects of sodium diacetate (SDA), sodium dehydroacetate (DHA-S), ε-polylysine hydrochloride (PLH), and nisin on RCM quality and bacterial community at concentrations of 3.0, 0.50, 0.30, and 0.50 g/kg, respectively. Major spoilage bacteria were isolated, and the inhibitory effects of these preservatives were studied, leading to the development of compound preservatives. TVC increased significantly during RCM preparation, with continuous increases in TVC and TVB-N levels throughout storage, reaching spoilage thresholds by day 5. Bacterial diversity decreased markedly, with *Brochothrix* and *Pseudomonas* dominating. SDA effectively inhibited TVC proliferation and TVB-N formation, maintaining bacterial diversity and reducing *Brochothrix* and *Pseudomonas* abundance while promoting the growth of lactic acid bacteria. Five spoilage bacteria strains were isolated, including *Serratia liquefaciens* B2107-1, a potent meat spoilage bacterium under refrigeration. PLH and SDA demonstrated significant inhibitory activity against this bacterium, with minimum inhibitory concentrations (MICs) of 0.175 and 0.400 mg/mL, respectively. Combining PLH and SDA at 1MIC + 3MIC exhibited a synergistic antimicrobial effect, maintaining RCM quality with reduced SDA usage. These findings demonstrate the significant potential of these preservatives in chilled, raw meat products.

## 1. Introduction

Mutton is a significant meat category globally, with extensive consumer demand and diverse production areas [1]. China ranks as a leading producer of mutton [2] due to its significant history in sheep farming for meat and substantial domestic mutton consumption [3]. According to the data from China’s National Bureau of Statistics, mutton production reached 5.18 million tons in 2024. The expansion of chain restaurants throughout China, combined with the emergence of the “lazy economy” phenomenon, has catalyzed the rapid growth in the raw pre-conditioned food sector [4], of which meat products are a key segment. Improvements in cold chain logistics technology have simultaneously fostered increasing consumer acceptance of chilled, raw pre-conditioned meat products.

Chilled, raw conditioned mutton (RCM) products contribute significantly to the advancement of the meat sheep industry by preserving freshness and nutritional content while also offering convenience and avoiding the detrimental impacts that conventional freezing methods can have on both the nutritional profile and flavor characteristics of meat products. However, the numerous processing steps necessary for RCM production increase the risk of microbial contamination. Additionally, the cutting process damages cellular structures and increases environmental exposure, thereby making the preservation of chilled RCM more challenging. Microbial spoilage is a significant constraint to the expansion and development of the chilled RCM industry [5].

The application of food preservatives is one of the most efficient methods for ensuring meat product quality. Various countries have implemented regulatory frameworks to regulate preservative usage in meat products [6]. According to China’s National Standard for Food Safety: Standard for the Use of Food Additives (GB 2760-2014) [7], four specific preservatives are approved for use in conditioned meat: sodium diacetate (SDA), sodium dehydroacetate (DHA-S), ε-polylysine hydrochloride (PLH), and nisin. These preservatives are subject to maximum permissible concentrations of 3.0, 0.50, 0.30, and 0.50 g/kg, respectively.

SDA and DHA-S represent chemicals that are characterized by cost-effectiveness and efficacy and have an extensive history of application in food products [8,9]. PLH and nisin are part of a select group of biological preservatives with practical food industry applications, and their utilization in food preservation continues to expand. When combined with other preservatives, both PLH and nisin demonstrate enhanced preservative effects compared with their individual applications, while also allowing for significant reductions in antimicrobial dosage requirements [10,11,12,13]. Furthermore, PLH and nisin are increasingly incorporated into biodegradable antimicrobial packaging materials, highlighting their antimicrobial properties [14,15,16]. Innovative antimicrobial components such as nisin have been formulated into edible coatings, demonstrating quality preservation effects on beef, chicken, and fish products, thereby extending their shelf life [17,18,19].

Currently, a significant gap exists in comprehensive research on preservation techniques for refrigerated conditioned meat. Zhang et al. [20] investigated the effects of packaging materials on conditioned fish quality. In the field of food additives, there is an emerging shift toward reducing chemical preservative usage by partially or completely substituting them with biological alternatives. An increasing number of studies are focusing on developing innovative natural or biological preservatives for meat, derived from plants and microorganisms [21,22,23]. While SDA, DHA-S, PLH, and nisin are authorized for conditioned meat preservation (GB 2760-2014) [7], their comparative preservation effectiveness has not been adequately examined. Furthermore, it is uncertain whether the biological preservatives PLH and nisin can achieve preservation efficacy comparable to chemical preservatives (SDA and DHA-S) when used within authorized concentration limits.

In this context, the preservation effects of SDA, DHA-S, PLH, and nisin on the quality of refrigerated RCM, as well as their inhibitory capacity against spoilage bacteria, are evaluated through our research, with optimized preservative application strategies yielding the development of favorable results. To achieve these objectives, we monitored changes in total viable count (TVC), total volatile basic nitrogen (TVB-N), and bacterial community diversity in RCM samples. We also isolated and characterized the predominant spoilage bacteria. Our investigation examined how these four preservatives influence the physicochemical properties and microbial ecology of RCM, as well as their inhibitory actions against key spoilage bacteria. Based on these experimental findings, effective preservative formulations were applied to refrigerated RCM for sensory evaluation and measurement of quality parameters, including TVC and TVB-N.

## 2. Materials and Methods

### 2.1. Conditioning of Mutton and Grouping of Preservative Tests

Fresh mutton, taken from the lean meat of the hind leg, was purchased at a local market in Shandong, China, close to the laboratory. It was then promptly transported to the lab, cleaned, naturally drained, and sliced into uniformly shaped pieces measuring 1–2 cm square. A 25 g portion was extracted to determine the TVC of fresh mutton before conditioning. The remaining meat pieces were subsequently divided into five distinct groups. For the RCM control without preservatives, basic ingredients, including salt (11.20 g/kg), monosodium glutamate (4.00 g/kg), and starch (16.00 g/kg), were incorporated and mixed thoroughly. For preservative treatment groups, different preservatives were added to the basic ingredients. The experimental preservative concentrations were 3.0 g/kg (SDA), 0.50 g/kg (DHA-S), 0.30 g/kg (PLH), and 0.50 g/kg (nisin). These concentrations represent the maximum permissible usage levels for different preservatives as per China’s National Standard for Food Safety: Standard for the Use of Food Additives (GB 2760-2014) [7]. Subsequently, all five RCM samples, with and without preservatives, were packed in polyethylene zip-lock food storage bags and stored at 4 °C for 9 d for evaluation of spoilage indicators, bacterial community diversity analysis, and spoilage bacteria isolation. All experiments were performed in triplicate.

### 2.2. Spoilage Indices Assay

The TVC assay was performed according to the method described by Li et al. [24]. Briefly, a 25 g meat sample was aseptically transferred under aseptic conditions into a sterile stomacher bag containing 225 mL of sterile saline, and homogenized for 3 min using an HX-4 beating sterile homogenizer (Shanghai Huxi Industrial Co., Ltd., Shanghai, China). Following homogenization, serial ten-fold dilutions of the homogenate were prepared using 0.9% saline. Each dilution was subsequently mixed with plate count agar (PCA) and incubated at 37 °C for 48 h. All counts were expressed as logarithms of colony-forming units per gram (log CFU/g).

TVB-N quantification was performed using the micro diffusion method according to the Chinese National Standard (GB 5009.228-2016) [25]. Specifically, a 10 g meat sample was homogenized with 100 mL of distilled water and centrifuged to extract the supernatant for analysis. In the inner chamber of the diffusion dish were placed 1 mL of 20 g/L boric acid and one drop of mixed indicator (composed of 1 mL of 1 g/L methyl red ethanol solution combined with 5 mL of 1 g/L bromocresol green ethanol solution). Meanwhile, 1 mL of the sample and l mL of saturated potassium carbonate (K_2_CO_3_) solution were added to the outer chamber of the diffusion dish, thoroughly mixed, and incubated at 37 °C for 2 h. Following incubation, 0.01 mol/L hydrochloric acid was used for titration until the solution became purple-red in color. TVB-N values were calculated according to the formula described by Wang et al. [26].

### 2.3. Bacterial Community Diversity

Genomic DNA was extracted from RCM samples using the EZNATM Mag-Bind Soil DNA Kit (Omega, M5635-02, Norcross, GA, USA), DNA integrity was assessed by 1% agarose gel electrophoresis, and DNA concentrations were measured using a Qubit^®^ 3.0 fluorometer (Q32866, Invitrogen, Carlsbad, CA, USA). The DNA solution concentration was adjusted to amplify the V3-V4 region of the specific 16S rRNA for PCR amplification with primers 341F (5′-CCTACGGGGNGGCWGCAG-3′) and 805R (5′-GACTACHVGGGTATCTAATCC-3′). The amplified products were confirmed by 2% agarose gel electrophoresis, and purified samples were quantified by Qubit^®^ 3.0 fluorometer (Q32866, Invitrogen, Carlsbad, CA, USA) and sent to Sangon Biotech (Shanghai) Co., Ltd. (Shanghai, China) for high-throughput sequencing. The paired-end (PE) reads obtained from sequencing were merged using overlapping regions, and sequences were filtered to obtain high-quality data. Subsequently, samples were subjected to cluster analysis of operational taxonomic units (OTUs). Based on OTUs, species diversity analysis and statistical analysis of bacterial community structures were performed [27,28].

### 2.4. Spoilage bacteria in RCM

#### 2.4.1. Isolation and Purification

Single bacterial colonies were randomly selected from the TVC assay plates of the RCM control stored on days 0, 1, 3, 5, 7, and 9. The primary criteria for strain selection were the diversity of colony morphologies, especially the shape, size, and color of the bacterial colonies. These colonies were subsequently purified by multiple streaking on Luria–Bertani (LB) agar plates.

#### 2.4.2. Classification and Identification

A well-developed colony exhibiting robust growth was selected for Gram staining and microscopic examination. The purified strains were sent to Sangon Biotech (Shanghai) Co., Ltd. (Shanghai, China) for 16S rDNA extraction, amplification, and sequencing. The resulting 16S rDNA sequences were submitted to the GenBank database (https://blast.ncbi.nlm.nih.gov/Blast.cgi) on 25 April 2024 for homologous comparative analysis. Multiple strains with high similarity were selected, and a phylogenetic tree was constructed and analyzed using Mega 11.0.13 software [29].

The morphological characteristics of strain B2107-1, which was subsequently identified as the most potent spoilage organism, were further examined using a scanning electron microscope (SU8100, Hitachi High-Technologies Corporation, Tokyo, Japan) at 3 kV voltage [30]. Biochemical characterization of strain B2107-1 was performed using the VITEK 2 compact automatic microbial identification instrument (bioMerieux, Marseille, France), along with a gram-negative bacterial identification card (GN) (bioMerieux, Marseille, France) [31,32]. Definitive species identification was achieved through comprehensive analysis that combined biochemical profiling, colony morphology assessment, Gram staining results, and molecular biological classification.

#### 2.4.3. Spoilage Potential Assessment

Isolated bacterial suspensions were inoculated into RCM samples at a final concentration of 10^5^ CFU/g. The inoculated RCM samples were packaged and stored at 4 °C for 9 days, with non-inoculated RCM serving as control. TVB-N content was measured to evaluate the spoilage capability of the five previously purified bacteria isolates [33].

### 2.5. Growth, Spoilage Enzyme Production, and Biogenic Amine (BA) Production of Spoilage Bacterium B2107-1 Under Refrigerated Conditions (4 °C)

A fresh culture of the strain, with a viable bacterial count of approximately 10^7^ CFU/mL during the early logarithmic phase, was transferred to LB broth medium at a 1% inoculation ratio and incubated at 4 °C. Growth was monitored by measuring OD_600_ values to generate a growth curve [34]. Additionally, extracellular protease and lipase activities were assessed according to the methods described by Dai et al. [35] and Li et al. [36]. Six BAs, including cadaverine, putrescine, tryptamine, tyramine, histamine, and spermidine, were quantified according to the standard method GB/T 5009.208-2016 [37].

### 2.6. Screening of Preservatives Against Spoilage Bacterium B2107-1 and Their Preservation Effects on RCM

Solutions of SDA, DHA-S, PLH, and nisin were prepared at concentrations of 30, 3, 0.3, and 0.03 mg/mL, respectively. The inhibitory activities of these preservatives were evaluated using the Oxford cup method, where 0.1 mL of each treatment solution was placed into cups and incubated at 37 °C for 48 h [38].

The OD_600_ of bacterial broth cultures containing varying concentrations of antimicrobial substances was measured to determine the minimum inhibitory concentration (MIC) and minimum bactericidal concentration (MBC) [10].

The RCM samples were inoculated with spoilage bacterium B2107-1 at a final concentration of 10^5^ CFU/g. These inoculated samples were packaged as control and stored at 4 °C for 9 days. The preservative treatment groups underwent identical processing but included the addition of appropriate amounts of either individual or compound preservatives. The antibacterial and preservation effects of different preservation strategies were evaluated through sensory evaluation (including color, odor, elasticity, and texture) [39], TVC measurement, and TVB-N content measurement.

### 2.7. Statistical Analysis

At least three samples were analyzed per group. The results were expressed as means ± standard deviation. Statistical analyses and data visualization were performed using GraphPad Prism 9.5.1 and Origin 2021 software. Statistical significance was assessed using Tukey’s multiple comparison tests, uncorrected Fisher’s LSD, *t*-tests, or nonparametric tests, with *p* < 0.05 considered statistically significant and *p* < 0.01 regarded as extremely statistically significant. Bioinformatic analyses, including Venn diagrams and principal coordinate analysis (PCoA) plot construction, were performed using OECloud tools, accessed at https://cloud.oebiotech.com on 12 October 2024. The phylogenetic tree was constructed using the Mega 11.0.13 software.

## 3. Results and Discussion

### 3.1. Spoilage Indices

TVC and TVB-N represent widely accepted indicators for evaluating the freshness of meat and fish products. According to the Chinese Agricultural Standard (NY/T 2073-2011) [40] and the Industry Group Standard (T/CCA 036-2024) [41], pre-processed meat products should maintain TVC levels below 6.0 log CFU/g and TVB-N values under 15 mg N/100 g. Measurements exceeding these thresholds indicate a decline in freshness and potential spoilage initiation, with meat considered to be spoiled and unsuitable for consumption when TVC surpasses 7.0 log CFU/g or TVB-N exceeds 20 mg N/100 g [42,43].

**Figure 1:**
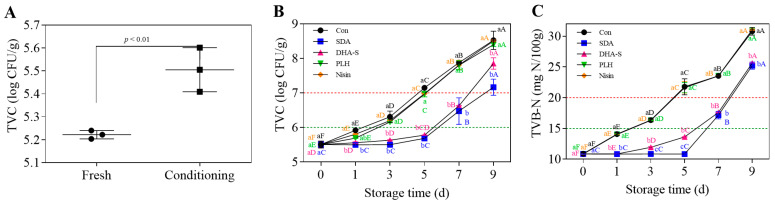
TVC of mutton before and after conditioning (**A**), and the effects of SDA, DHA-S, PLH, and nisin treatments on TVC (**B**) and TVB-N (**C**) of RCM stored at 4 °C for 9 d. ^a–c^ Different lowercase letters indicate a significant difference between groups at the same storage time (*p <* 0.05); ^A–F^ different uppercase letters denote a significant difference across the storage time (d) within a single group (*p <* 0.05). TVC: total viable count; TVB-N: total volatile basic nitrogen; Con: control; SDA: sodium diacetate; DHA-S: sodium dehydroacetate; PLH: ε-polylysine hydrochloride; RCM: raw conditioned mutton.

The conditioning process significantly (*p* < 0.01) increased the initial TVC levels in the RCM compared with fresh mutton (Figure 1A). This finding underscores the necessity of preservation treatment to maintain RCM quality and safety throughout its shelf life. Increased TVC values resulting from microbial contamination are a primary factor contributing to meat spoilage [44].

As shown in Figure 1B,C, both TVC and TVB-N values of the RCM control increased significantly (*p* < 0.01). After storage at 4 °C for 3 days, the mean TVC and TVB-N values reached 6.31 log CFU/g and 16.36 mg N/100 g, respectively, exceeding the thresholds indicating standard freshness; by day 5, parameters increased to 7.15 log CFU/g and 21.77 mg N/100 g, signifying substantial spoilage. The proliferation of spoilage bacteria and the significant increase in TVB-N values can notably affect nutritional quality and pose potential foodborne illness risks [45]. As illustrated in Figure 1B,C, compared with the RCM control, SDA and DHA-S significantly suppressed the rise in TVC and TVB-N values of RCM (*p* < 0.01), whereas PLH and nisin treatments did not reduce these indices (*p* > 0.05). Evidently, even at the maximum permissible concentrations specified in GB 2760-2014 [7], using these biological preservatives alone is not sufficient to ensure the effective preservation of RCM. The concept of completely substituting chemical preservatives with biological alternatives without implementing complementary strategies is impractical. The nonlinear regression analysis was performed on the data presented in Figure 1B, with the results summarized in Table 1. The groups Con, PLH, and Nisin were curve fitted using the exponential (Malthusian) growth model based on the exponential growth curves equation. The R^2^ values of 0.9840, 0.9896, and 0.9926 respectively indicate a good fit for all three groups, demonstrating that the TVC increased exponentially with storage time. The specific growth rates were determined to be 0.04835, 0.04923, and 0.04936, respectively. In contrast, the groups SDA and DHA-S could not be fitted using models related to growth curves, including the commonly employed modified Gompertz model. This model is typically applied to microbial growth curves to describe the lag phase and early exponential phase; however, it was unsuitable for these groups. Instead, a quadratic equation was used for nonlinear fitting of the groups SDA and DHA-S, yielding R^2^ values of 0.9320 and 0.9843 respectively, which also indicates a satisfactory fit. Predictions of shelf life based on the fitted equations showed that the SDA and DHA-S treatments could extend the shelf life by 4.26 days and 3.75 days, respectively, whereas the PLH and nisin treatments extended the shelf life by less than half a day. Moreover, SDA was more effective than DHA-S, showing a significant difference (*p* < 0.05) in their effect on TVB-N values of RCM on days 3 and 5 (Figure 1C). The TVC and TVB-N values of RCM treated with SDA and stored at 4 °C for 5 days showed no significant difference from values on day 0 (Figure 1B,C), with both freshness and severe spoilage thresholds delayed by approximately 4 days. According to GB 2760-2014 [7], the maximum allowable addition of SDA in RCM is six times that of DHA-S. This significant difference is likely the primary reason for SDA’s superior preservation effect compared with DHA-S. Additionally, this may be due to the varying sensitivity of RCM spoilage bacteria to different preservatives.

### 3.2. Bacterial Community Diversity by 16S rDNA Sequencing

#### 3.2.1. Sequencing Data and Alpha Diversity Analysis

To investigate and compare the effects of various preservatives on the bacterial community of RCM, we first investigated the bacterial community evolution in RCM samples stored at 4 °C for 1 to 9 d. Subsequently, the effects of four preservatives on the bacterial diversity in RCM at a critical spoilage time point, when the samples began to deteriorate and bacterial community structure underwent significant changes, were analyzed. This time point was subsequently confirmed as day 5 of storage. High-throughput sequencing results revealed a total of 863,214 effective gene sequence bands, with an average length of 428 bp from 27 samples. OTU clustering of non-repetitive sequences was performed based on 97% similarity, and the coverage index (Table 2) for all samples exceeded 99%, indicating that the sequencing results could accurately represent the microbial diversity within the samples.

The ACE and Chao indexes based on Alpha diversity analysis represent the richness of community species, with values increasing as species richness rises. The Simpson and Shannon indexes represent community species diversity, with the Simpson index decreasing as community diversity increases, while the Shannon index exhibits the opposite trend [46]. Alpha diversity measurements are presented in Table 2. For the RCM control, the OTU count generally decreased over time, indicating a progressive reduction in bacterial community abundance during storage. Similar trends were observed from the ACE and Chao indexes, indicating that the richness of community species significantly decreased on day 5 (*p* < 0.05). Compared with day 3, the Simpson index increased by 57.89% and the Shannon index decreased by 52.43% on day 5, indicating a decrease in bacterial community diversity over this period. The period from day 3 to day 5 represents a critical period of rapid change in bacterial community abundance and diversity, consistent with previous findings where TVC and TVB-N exceeded raw meat spoilage thresholds on day 5 (Figure 1B,C). This indicates that, when RCM samples reach spoilage levels on day 5, significant increases in viable bacterial count occur, accompanied by substantial changes in bacterial community structure and an extensive production of protein volatile decomposition products. Delaying this transformation is crucial for maintaining the RCM quality. Therefore, the effects of the four preservatives on bacterial flora on day 5 were investigated and compared. SDA treatment significantly slowed the decline in bacterial community abundance (*p* < 0.05), whereas DHA-S, PLH, and nisin treatments showed no significant effects (*p* > 0.05).

#### 3.2.2. Analysis of Bacterial Community Structure and Beta Diversity

**Figure 2:**
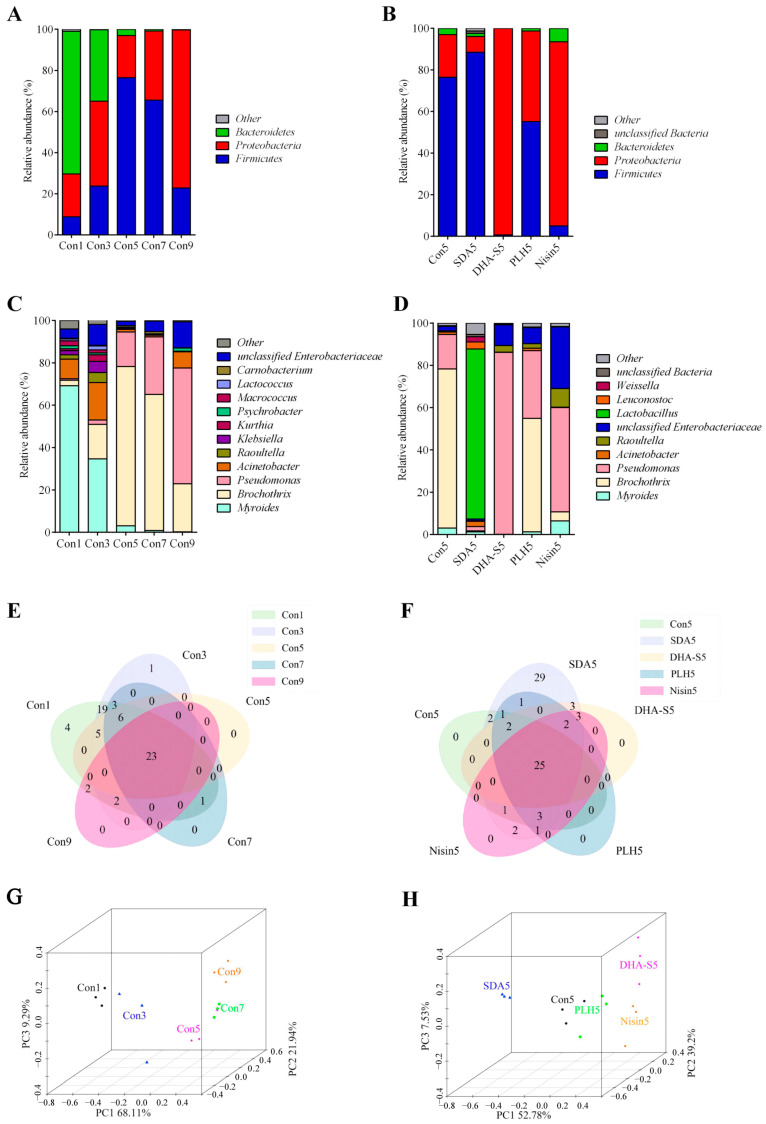
Relative abundance of bacterial community members at the phylum (**A**,**B**) and genus (**C**,**D**) levels, Venn diagrams (**E**,**F**), and PCoA 3D plots (**G**,**H**) at genus level in RCM stored at 4 °C. Con1, Con3, Con5, Con7, and Con9 represent RCM controls stored at 4 °C for 1, 3, 5, 7, and 9 days, respectively; SDA5, DHA-S5, PLH5, and Nisin5 denote samples treated with SDA, DHA-S, PLH, and nisin, respectively, and stored at 4 °C for 5 days. Each sample is analyzed in triplicate. SDA: sodium diacetate; DHA-S: sodium dehydroacetate; PLH: ε-polylysine hydrochloride; RCM: raw conditioned mutton.

Based on the OTU analysis, the data from 27 samples were classified and analyzed at both phylum and genus taxonomic levels. The relative abundance of bacterial communities at the phylum level are illustrated in Figure 2A,B, while the genus level distribution is presented in Figure 2C,D.

*Bacteroidetes*, *Proteobacteria*, *Firmicutes*, and other unclassified bacteria constituted the predominant bacterial community. On day 1, the proportions of *Bacteroidetes*, *Proteobacteria*, and *Firmicutes* in the RCM control were 69.39%, 20.82%, and 8.84%, respectively. Throughout the storage period, the proportion of *Bacteroidetes* decreased to 34.78% and 3.08% on days 3 and 5, respectively, representing reductions of 34.61% (*p* < 0.01) and 66.31% (*p* < 0.01) compared with day 1. The proportions of *Firmicutes* and *Proteobacteria* increased dynamically compared with day 1, reaching their highest levels on days 5 (*p* < 0.01) and 9 (*p* < 0.01), respectively (Figure 2A). On day 5, the effects of PLH, nisin, DHA-S, and SDA treatments on the bacterial species proportions at phylum level demonstrated differences. Only SDA treatment reduced the proportion of *Proteobacteria* (*p* > 0.05), while the other three preservatives increased its relative abundance (Figure 2B), and the increases induced by nisin and DHA-S were significant (*p* < 0.05).

As depicted in Figure 2C, on day 1 the predominant genera in the RCM control included *Myroides* (69.22%), *Brochothrix* (2.60%), *Pseudomonas* (0.69%), *Acinetobacter* (9.26%), *Raoultella* (2.07%), and unclassified *Enterobacteriaceae* (4.33%). Throughout the storage period, the abundance of *Myroides* decreased to 3.08% (*p <* 0.05), while *Brochothrix* and *Pseudomonas* increased respectively to 75.28% (*p <* 0.05) and 16.28% (*p* > 0.05) by day 5. By day 9, the abundance of *Pseudomonas* further increased to 54.67% (*p <* 0.05), while *Brochothrix* decreased to 22.61% (*p* > 0.05). *Myroides* represents the dominant bacterial flora in fresh RCM, while *Brochothrix* and *Pseudomonas* constitute the dominant bacterial groups in spoiled RCM.

As illustrated in Figure 2D, SDA, DHA-S, PLH, and nisin treatments all reduced the abundance of *Brocothrix* on day 5. Among these, the inhibitory effects of SDA and DHA-S against *Brocothrix* were extremely significant (*p* < 0.01). SDA and DHA-S, both organic acid preservatives, exert bacteriostatic effects through multi-target mechanisms, showcasing broad-spectrum antibacterial properties. The chemical structures of these two compounds share similarities, as they both contain acetate ions, resulting in numerous resemblances in their mechanisms of action. Both can disrupt the integrity of cell membranes, leading to leakage of cellular contents; induce acidification of cells through the permeation of acetic acid; and disrupt enzymatic activity and metabolic balance within cells. Additionally, DHA-S can directly interfere with DNA synthesis and chelate metal ions [47]. Overall, at the same concentration, DHA-S exhibits stronger antimicrobial activity than SDA; however, SDA is considered safer with more lenient regulatory restrictions regarding its application range and dosage limits. Regarding the abundance of *Pseudomonas*, which has been reported as a dominant organism in various types of spoiled meat [48,49,50,51], only SDA treatment can reduce its relative abundance, while the other three preservatives actually increase its relative abundance, and the increasing effect of DHA-S is significant (*p* < 0.05). SDA treatment also significantly increased the relative abundance of *Lactobacillus* from 0.01% to 80.37% (*p* < 0.01), *Leuconostoc* from 0.01% to 3.33% (*p* > 0.05), and *Weissella* from 0.14% to 2.52% (*p* > 0.05). These significantly increased bacteria belong to LAB. LAB is beneficial to the biological preservation of meat. They produce metabolites such as bacteriocins and lactic acid that control spoilage bacteria in meat products, thereby enhancing the sensory quality, nutritional value, and shelf life of meat products [52]. Figure 2D also indicates that nisin treatment increased the proportion of gram-negative bacteria such as *Enterobacteriaceae* (*p* < 0.01) and *Pseudomonas* (*p* > 0.05) in the microbial community. These categories of gram-negative bacteria are crucial spoilage organisms in meat products. This result provides a key explanation for nisin’s inability to inhibit the increase of TVC in RCM, as shown in Figure 1B.

Figure 2E,F show the Venn diagrams at the genus level, which corroborate the bacterial community diversity observed in the Alpha diversity analysis. PCoA effectively demonstrates the Beta diversity among communities, with shorter distances between samples indicating greater similarity in species composition and structure [53]. Figure 2G,H show the PCoA analysis at the genus level, while Table 3 and Table 4 reveal the spearman correlation of the samples. The RCM control samples from days 1 and 3 are relatively close to each other, suggesting minor alterations in community composition during this period. In contrast, the distance between samples from days 3 and 5 is relatively far, suggesting a substantial change in community structure. Notably, the sample from SDA5 is closely positioned to Con1 but distant from Con5, suggesting that the Beta diversity of RCM after SDA treatment is more similar to that of fresher samples.

#### 3.2.3. Differential Species Analysis of Bacterial Community

**Figure 3:**
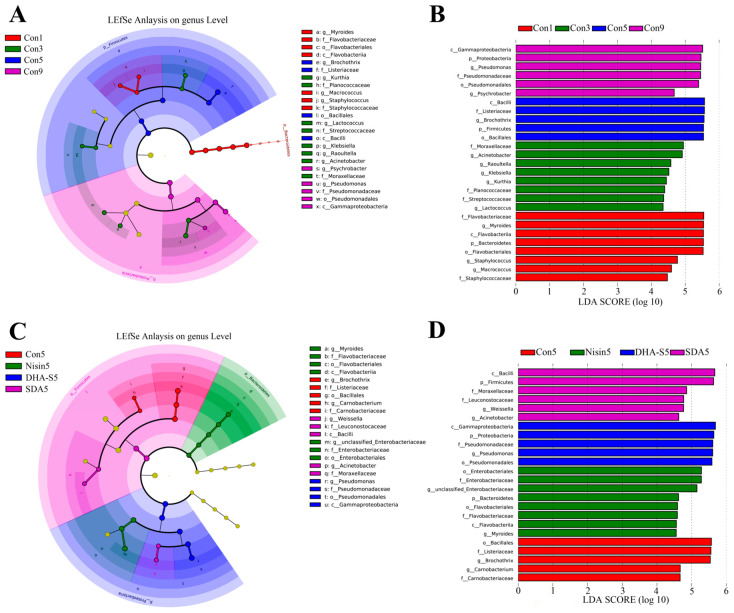
Cladograms (**A**,**C**) and LDA scores (**B**,**D**) based on LEfSe analysis (at genus level).

**Figure 4:**
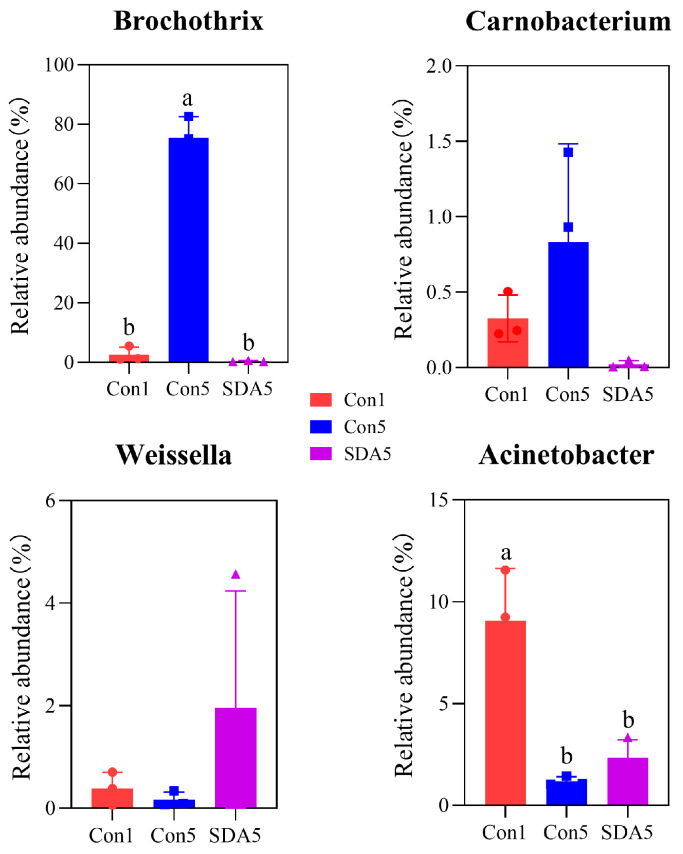
Significance analysis of differential species. ^a,b^ Different lowercase letters indicate a significant difference (*p <* 0.05).

The analysis using LEfSe revealed that, at the genus level, the differentially abundant bacterial genera in the control group Con1 were *Myroides*, *Staphylococcus*, and *Macrococcus*. The differentially abundant bacteria for group Con3 were *Acinetobacter*, *Raoultella*, *Klebsiella*, *Kurthia*, and *Lactococcus*. For group Con5, the differentially abundant bacteria were *Brochothrix*. In group Con9, the differentially abundant bacteria were *Pseudomonas* and *Psychrobacter* (Figure 3A,B). The LEfSe analysis of the preservative treatment experiment showed that the differentially abundant bacteria for group Con5 were *Brochothrix* and *Carnobacterium*, while for SDA-treated group, they were *Weissella* and *Acinetobacter* (Figure 3C,D). Further analysis of the differentially abundant bacteria between the Con5 group and the SDA-treated group revealed that the SDA treatment inhibited the increase in relative abundance of *Brochothrix* (*p* < 0.05) and *Carnobacterium* in RCM, while also preventing the decrease in relative abundance of *Weissella* and *Acinetobacter*, as shown in Figure 4.

### 3.3. Isolation of Spoilage bacteria and Comparison of Their Spoilage Potential

**Figure 5:**
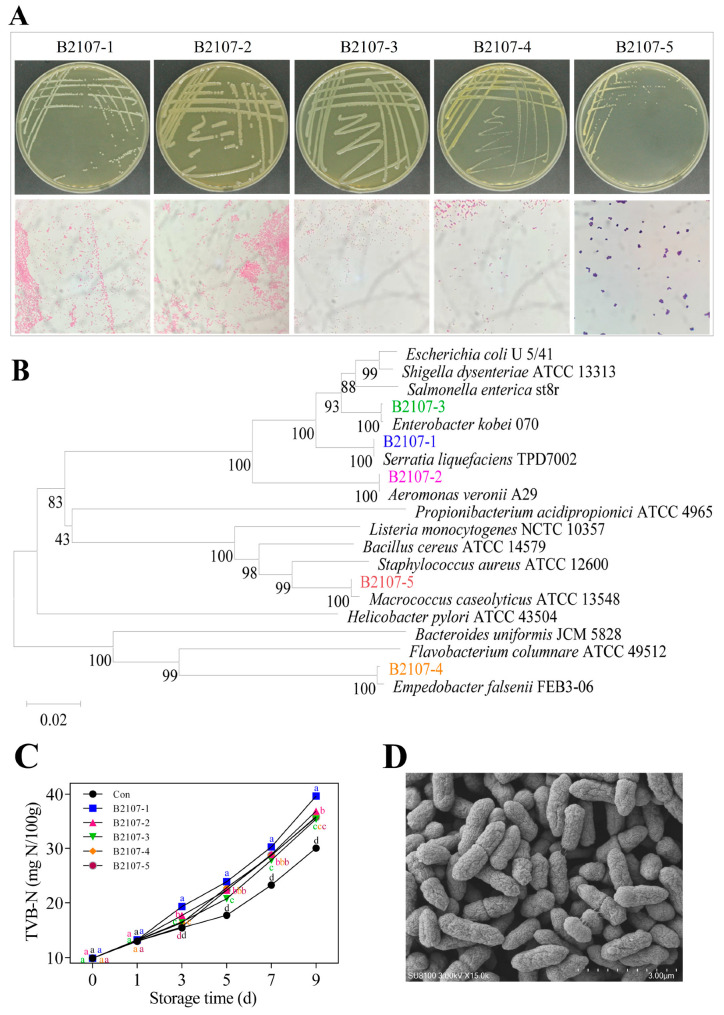
Colony morphology and Gram staining (**A**), Phylogenetic tree (**B**) of five strains of spoilage bacteria labeled B2107-1 through B2107-5 (37 °C, 24 h), their effect on TVB-N (**C**) of RCM stored at 4 °C, and SEM morphology of strain B2107-1 (**D**). ^a–d^ Different lowercase letters in Figure 5C denote significant differences between groups at the same storage time (*p* < 0.05). TVB-N: total volatile basic nitrogen; RCM: raw conditioned mutton; SEM: scanning electron microscopy.

Initially, strains were isolated from the RCM control that had been stored for 0 days, and colonies with differences were streaked for purification and further observation. Subsequently, the TVC detection plates of the RCM control that had been stored for 1 day were examined and compared with those from day 0, selecting strains with varying colony morphologies. This process was repeated consistently until day 9. Colonies were randomly selected from each sample. If they were suspected to represent different strains based on colony morphology, they were chosen for further purification and examination. Finally, five bacterial strains were isolated from the refrigerated RCM control stored at 4 °C, and labeled B2107-1 through B2107-5. Colony morphology and cellular morphology of these strains were observed through plate streaking purification and Gram staining, respectively (Figure 5A). All strains exhibited circular single colonies with well-defined edges; smooth, moist, glossy surfaces; and an opaque cream-like appearance when grown on LB plates. Strains B2107-1, B2107-4, and B2107-5 formed raised colonies, while strains B2107-2 and B2107-3 exhibited slightly elevated centers. Minor variations in colony color and size were also observed. The strain colors were as follows: B2107-1 was milky white, B2107-2 was grayish white, B2107-3 was milky white, B2107-4 was yellowish brown, and B2107-5 was grayish white. After 24 h of cultivation, the colony diameters measured 1.65 mm for B2107-1, 2.65 mm for B2107-2, 3.05 mm for B2107-3, 0.71 mm for B2107-4, and 0.56 mm for B2107-5. Gram staining revealed that strains B2107-1 to B2107-4 were gram-negative bacilli, arranged individually or in pairs, while strain B2107-5 was a gram-positive cocci, appearing in pairs, tetrads, or clusters.

The 16S rDNA sequences of the five strains were aligned against the GenBank database to construct a phylogenetic tree (Figure 5B). The strains B2107-1 through B2107-5 exhibited sequence similarities of 100%, 100%, 99.79%, 99.65%, and 100% with *Serratia liquefaciens* TPD7002, *Aeromonas Veronii* A29, *Enterobacter kobei* 070, *Empedobacter falsenii* FEB3-06, and *Macrococcus caseolyticus* ATCC 13548, respectively, positioning on the same branch of the tree. After inoculation with these five bacterial strains, the TVB-N values in the RCM increased across all samples. Notably, strain B2107-1 caused the most significant increase in TVB-N (Figure 5C). This suggests that all isolated strains were indeed spoilage bacteria, with strain B2107-1 exhibiting particularly detrimental effects on chilled fresh RCM quality. To further validate the identity of strain B2107-1, its morphology was observed using scanning electron microscopy (Figure 5D), its biochemical properties were systematically analyzed (Table 5), and the biochemical characteristics were compared with those reported by Michail et al. [54]. Based on these comprehensive analyses, strain B2107-1 was identified as *Serratia liquefaciens*.

### 3.4. Characteristics of Serratia Liquefaciens B2107-1 at 4 °C

**Figure 6:**
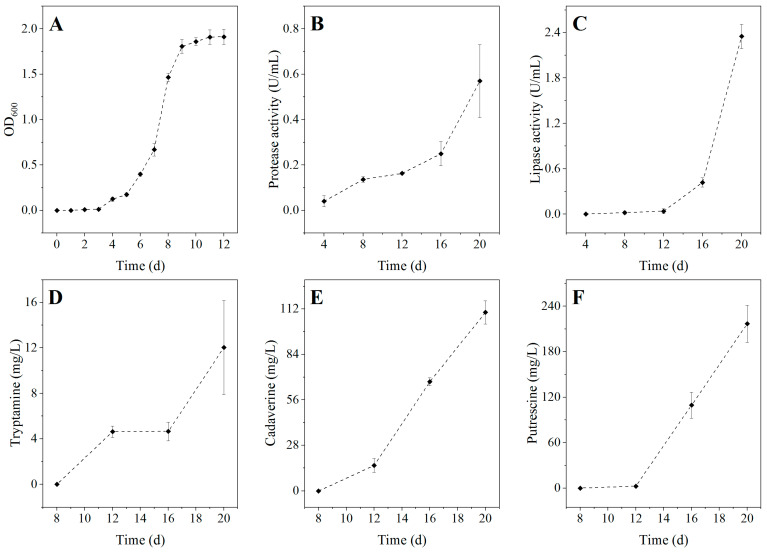
Growth curve (**A**), extracellular protease activity (**B**), extracellular lipase activity (**C**), and BA production (**D**–**F**) of *Serratia liquefaciens* B2107-1 at 4 °C. BA: biogenic amine.

Figure 6A illustrates the growth curve of *Serratia liquefaciens* B2107-1 at 4 °C. The data reveal that the OD_600_ value for this bacterium can reach up to 1.911 at 4 °C, highlighting its remarkable ability to proliferate even under refrigeration temperatures. Further investigation of its spoilage potential includes the evaluation of extracellular enzyme production. As illustrated in Figure 6B,C, *Serratia liquefaciens* B2107-1 exhibits activity in secreting both protease and lipase at 4 °C. These activities increase over time. The extracellular protease and lipase secreted by this strain facilitate the degradation of proteins and lipids in refrigerated RCM, accelerating its spoilage process [55]. An analysis of biogenic amine (BA) production by *Serratia liquefaciens* B2107-1 at 4 °C was also conducted. The results show that tyramine, histamine, and spermidine were not detected. However, the bacterium was found to produce cadaverine, putrescine, and tryptamine. Among these, tryptamine is highly toxic, while cadaverine and putrescine, despite their lower toxicity, are the primary contributors to the foul odor associated with spoiled meat [56]. Details are presented in Figure 6D–F. Notably, BA production gradually increased with extended incubation periods, with cadaverine and putrescine being predominant over tryptamine. These spoilage characteristics are directly correlated with the bacterium’s capacity to cause meat spoilage [57].

The main pathways through which *Serratia liquefaciens* B2107-1 decomposes proteins to generate cadaverine, putrescine, and tryptamine are proposed as follows: *Serratia liquefaciens* B2107-1 demonstrates not only extracellular protease activity but also tests positive for lysine decarboxylase and ornithine decarboxylase in qualitative assays (Table 5). The extracellular protease secreted by B2107-1 degrades proteins into amino acids such as lysine, ornithine, glutamic acid, arginine, and tryptophan. Lysine is subsequently converted into cadaverine through the action of lysine decarboxylase produced by B2107-1. Glutamic acid and arginine can be transformed into ornithine, which is subsequently metabolized by ornithine decarboxylase from B2107-1 to produce putrescine. Tryptophan is converted into tryptamine by tryptophan decarboxylase, and may further degrade to produce indole, another malodorous byproduct of protein degradation.

### 3.5. Potential Preservatives Against Serratia Liquefaciens B2107-1 and Its Preservation Effect on RCM

*Serratia liquefaciens* has been insufficiently studied in food research contexts. While not classified as a foodborne pathogen, it has been identified as a predominant spoilage organism in various refrigerated food products. Studies by Machado et al. [58,59] have highlighted that *Serratia liquefaciens* represents a dominant psychrotrophic microorganism in cold raw milk, exhibiting significant activity in producing heat-resistant protein hydrolase and significant spoilage potential. Additionally, Salgado et al. [60] identified heat-stable lipase secreted by *Serratia liquefaciens* that exhibited substantial spoilage potential in milk. Begrem et al. [61] have further reported that *Serratia liquefaciens* is predominant in cold-smoked salmon. Therefore, developing effective antibacterial agents against *Serratia liquefaciens* is essential.

**Figure 7:**
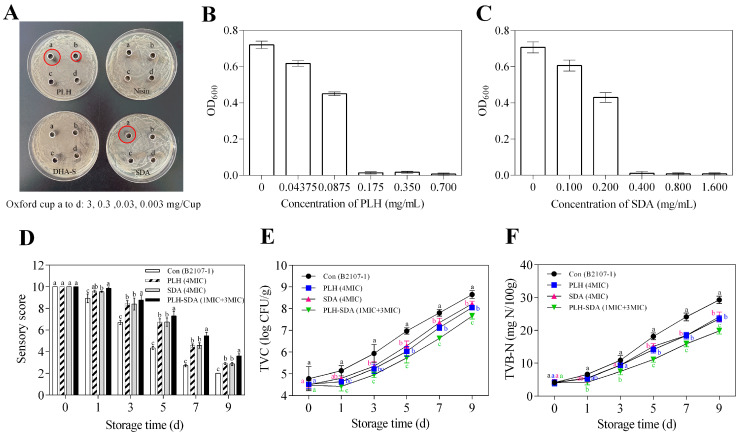
Screening of antibacterial agents against *Serratia liquefaciens* B2107-1 (**A**), MIC values of PLH and SDA against *Serratia liquefaciens* B2107-1 (**B**,**C**), sensory scores (**D**), TVC (**E**), and TVB-N (**F**) of RCM stored at 4 °C for 9 days. The red circle in (**A**) highlights the antibacterial zone. ^a–c^ Different lowercase letters in (**D**–**F**) indicate significant differences between groups at the same storage time (*p <* 0.05). Con (B2107-1): RCM control inoculated with *Serratia liquefaciens* B2107-1; PLH (4MIC): RCM inoculated with *Serratia liquefaciens* B2107-1 and treated with 4MIC PLH; SDA (4MIC): RCM inoculated with *Serratia liquefaciens* B2107-1 and treated with 4MIC SDA; PLH-SDA (1MIC + 3MIC): RCM inoculated with *Serratia liquefaciens* B2107-1 and treated with a combination of MIC PLH and 3MIC SDA. MIC: minimum inhibitory concentration; SDA: sodium diacetate; PLH: ε-polylysine hydrochloride; RCM: raw conditioned mutton.

The antimicrobial activities of SDA, DHA-S, PLH, and nisin against *Serratia liquefaciens* B2107-1 were evaluated and the results are shown in Figure 7A. Notably, at concentrations below or equal to 3 mg/Oxford cup, nisin and DHA-S exhibited negligible inhibitory zones, indicating minimal antibacterial efficacy. In contrast, PLH demonstrated substantial inhibitory activity, forming clear zones with diameters of 13 mm at 0.3 mg/Oxford cup and 21 mm at 3 mg/Oxford cup. SDA, on the other hand, showed no observable inhibition at the lower concentration but formed a significant inhibition zone, with a diameter of approximately 22 mm at 3 mg/Oxford cup, comparable in size to that of PLH at the equivalent dosage. Meanwhile, a key distinction was observed between the inhibition patterns of PLH and SDA: the former produced a transparent zone entirely devoid of bacterial growth, whereas the latter’s inhibition area exhibited smaller, sparsely distributed colonies. This observation led to the preliminary conclusion that PLH exhibits superior antibacterial efficacy against *Serratia liquefaciens* B2107-1 compared with SDA, underscoring its potential as a more effective inhibitor.

To quantitatively evaluate the antibacterial efficacy of PLH and SDA, their MIC and MBC were determined for B2107-1. The MIC values were established at 0.175 mg/mL for PLH and 0.400 mg/mL for SDA (Figure 7B,C). The corresponding MBC values were 175 μg/mL for PLH and 4000 μg/mL for SDA (Table 6). Although SDA effectively delayed RCM spoilage, its application at higher concentrations could negatively impact the product’s color and impart an undesirable sour odor. Therefore, combining these two preservatives may provide an enhanced strategy for improving the safety and quality of chilled RCM.

*Serratia liquefaciens* B2107-1 was inoculated into RCM as a control, and antimicrobial treatments with PLH (4MIC), SDA (4MIC), and PLH-SDA (1MIC + 3MIC) were applied to evaluate their preservative efficacy. The quality assessment results of RCM are presented in Figure 7D–F. The sensory quality, TVC values, and TVB-N values of RCM were all affected by the interaction between treatment method and storage time. During the 9-day storage period, sensory evaluation scores decreased, while TVC and TVB-N values rose. Both PLH and SDA at 4MIC significantly delayed changes in these quality indicators when applied individually (*p* < 0.05). However, the concentration of PLH used at 4MIC exceeds the permissible legislative limit of 0.3 g/kg, rendering it impractical for use. The advantage of SDA lies in its higher upper limit for use in pre-conditioned meat products (3 g/kg), with the concentration used (4MIC) being well below its upper limit. Additionally, when PLH and SDA are combined in a 1MIC + 3MIC ratio, they demonstrate significant synergistic effects, surpassing their individual effects (*p* < 0.05). These findings align with previous reports indicating that PLH and SDA demonstrate synergistic effects when combined with other antimicrobials [10,11,12,13]. Moreover, this combination ratio does not exceed the upper limit for the use of preservative blends, thereby offering excellent practical application value. According to the literature [62], the primary antibacterial mechanism of PLH involves its direct attachment to the microbial cell membrane, resulting in membrane disruption, as well as its penetration into the microorganism, where it inhibits protein synthesis and induces DNA damage. Figure 7B and Table 6 indicate that the MIC and MBC values of PLH against *Serratia liquefaciens* B2107-1 are identical, suggesting that its damage to bacterial cells is likely irreversible and lethal. In contrast, SDA primarily influences microbial metabolism through the acidification effect of acetic acid, thereby suppressing microbial growth [47]. The MIC and MBC concentrations of SDA against *Serratia liquefaciens* B2107-1 differ by a factor of ten (Figure 7C, Table 6), indicating that lower concentrations of SDA merely exert an inhibitory effect. There is a significant difference in the action targets of these two agents on microorganisms. The synergistic preservation effect observed when PLH and SDA are combined in RCM may be attributed to their potential synergistic antibacterial activity.

## 4. Conclusions

This study highlights the application of food preservatives in the chilled, raw conditioned meat industry, in accordance with the Chinese National Standard. This preservation scheme for chilled, raw conditioned meat, based on food preservatives, is legal, compliant, and of significant practical value. Furthermore, this study addresses a gap in the current literature. The pretreatment process significantly increases the initial bacterial viable counts in RCM products. The products are highly susceptible to deterioration during refrigeration and have a limited shelf life. However, the addition of SDA within the limited concentration can extend the shelf life by approximately 4 days. The SDA treatment also significantly decelerated the reduction in bacterial community abundance and diversity of RCM, substantially decreasing the relative abundance of spoilage bacteria *Brochothrix* and *Pseudomonas*, while increasing the population of beneficial lactic acid bacteria (LAB) in the product. In addition, a strain of *Serratia liquefaciens* B2107-1 was isolated from RCM. It is a strain that can easily cause meat corruption. Both PLH and SDA can significantly inhibit the growth of this bacterium, with MICs of 0.175 mg/mL and 0.400 mg/mL, respectively. When PLH and SDA were applied in combination at concentrations of 1MIC + 3MIC for the preservation of RCM, these two food preservatives exhibited synergistic antibacterial and preservation effects, reducing the required amount of the chemical preservative SDA while maintaining the high quality of RCM. This indicates their significant potential for application in the preservation of chilled, raw conditioned meat products.

Future research should focus on elucidating the synergistic antibacterial mechanism of PLH and SDA against *Serratia liquefaciens* and their synergistic preservation mechanism in conditioned meat products, as well as further optimizing the compound formulation. This is essential for promoting their widespread application in the food industry.

## Figures and Tables

**Table 1:** Nonlinear regression analysis of TVC values.

Sample	Kinetic Equation	*R* ^2^	PV/d
Con	y = 5.559*e*^0.04835t^	0.9840	1.57
SDA	y = 5.533 − 0.1143t + 0.03330t^2^	0.9320	5.83
DHA-S	y = 5.612 − 0.1891t + 0.04917t^2^	0.9843	5.32
PLH	y = 5.434*e*^0.04923t^	0.9896	2.01

Note: The lowercase letters ‘y’ and ‘t’ denote the TVC value and storage time, respectively. ‘PV’ represents the predicted shelf life based on a TVC threshold of less than 6.0 CFU/g.

**Table 2:** Statistics of Alpha diversity indices.

Sample	OTU	Coverage/%	ACE	Chao	Simpson	Shannon
Con1	146	99.86 ± 0.07	202.06 ± 41.09 ^a^	185.22 ± 28.04 ^a^	0.36 ± 0.21	1.85 ± 0.55 ^a^
Con3	111	99.88 ± 0.08	207.63 ± 70.12 ^a^	167.82 ± 54.18 ^a^	0.38 ± 0.16	1.62 ± 0.28 ^ab^
Con5	46	99.94 ± 0.02	85.45 ± 10.40 ^bB^	72.98 ± 10.23 ^bB^	0.60 ± 0.08	0.88 ± 0.14 ^b^
Con7	46	99.94 ± 0.02	101.14 ± 31.76 ^ab^	74.53 ± 9.40 ^b^	0.50 ± 0.03	0.95 ± 0.05 ^b^
Con9	38	99.96 ± 0.02	67.00 ± 35.79 ^b^	61.33 ± 30.92 ^b^	0.39 ± 0.06	1.25 ± 0.18 ^ab^
SDA5	156	99.79 ± 0.06	199.73 ± 27.75 ^A^	184.73 ± 38.12 ^A^	0.51 ± 0.22	1.40 ± 0.39
DHA-S5	37	99.94 ± 0.00	87.93 ± 13.14 ^B^	68.73 ± 15.14 ^B^	0.75 ± 0.17	0.55 ± 0.35
PLH5	48	99.95 ± 0.02	69.42 ± 18.67 ^B^	65.62 ± 18.47 ^B^	0.42 ± 0.05	1.22 ± 0.27
Nisin5	48	99.93 ± 0.02	93.51 ± 42.05 ^B^	73.94 ± 28.73 ^B^	0.36 ± 0.09	1.38 ± 0.22

Note: Con1, Con3, Con5, Con7, and Con9 represent RCM controls stored at 4 °C for 1, 3, 5, 7, and 9 d, respectively; SDA5, DHA-S5, PLH5, and Nisin5 denote RCM samples treated with SDA, DHA-S, PLH, and nisin, respectively, then stored at 4 °C for 5 d. Each sample was analyzed in triplicate. ^a,b^ Different lowercase letters indicate a significant difference in the control group over time (*p <* 0.05). ^A,B^ Different uppercase letters denote a significant difference in each preservative treatment and Con5. Con: control; SDA: sodium diacetate; DHA-S: sodium dehydroacetate; PLH: ε-polylysine hydrochloride; RCM: raw conditioned mutton.

**Table 3:** Spearman correlation of samples Con1 to Con9 at the genus level.

	Con1-1	Con1-2	Con1-3	Con3-1	Con3-2	Con3-3	Con5-1	Con5-2	Con5-3	Con7-1	Con7-2	Con7-3	Con9-1	Con9-2	Con9-3
Con1-1	1.00	0.55	0.62	0.83	0.44	0.89	0.78	0.69	0.74	0.62	0.74	0.62	0.73	0.73	0.77
Con1-2	0.55	1.00	0.92	0.38	0.80	0.48	0.07	0.04	0.05	−0.10	0.04	0.00	−0.09	0.01	0.02
Con1-3	0.62	0.92	1.00	0.33	0.76	0.46	0.07	0.03	0.03	−0.04	0.05	0.01	0.02	0.14	0.10
Con3-1	0.83	0.38	0.33	1.00	0.20	0.94	0.76	0.66	0.73	0.52	0.68	0.45	0.64	0.54	0.63
Con3-2	0.44	0.80	0.76	0.20	1.00	0.36	0.07	−0.07	0.08	−0.09	0.08	0.09	−0.04	0.02	0.01
Con3-3	0.89	0.48	0.46	0.94	0.36	1.00	0.81	0.72	0.79	0.60	0.76	0.55	0.68	0.58	0.70
Con5-1	0.78	0.07	0.07	0.76	0.07	0.81	1.00	0.93	0.96	0.92	0.97	0.88	0.92	0.82	0.93
Con5-2	0.69	0.04	0.03	0.66	−0.07	0.72	0.93	1.00	0.94	0.93	0.95	0.89	0.81	0.74	0.89
Con5-3	0.74	0.05	0.03	0.73	0.08	0.79	0.96	0.94	1.00	0.90	0.98	0.89	0.87	0.72	0.88
Con7-1	0.62	−0.10	−0.04	0.52	−0.09	0.60	0.92	0.93	0.90	1.00	0.95	0.95	0.90	0.83	0.94
Con7-2	0.74	0.04	0.05	0.68	0.08	0.76	0.97	0.95	0.98	0.95	1.00	0.94	0.90	0.80	0.93
Con7-3	0.62	0.00	0.01	0.45	0.09	0.55	0.88	0.89	0.89	0.95	0.94	1.00	0.83	0.77	0.89
Con9-1	0.73	−0.09	0.02	0.64	−0.04	0.6	0.92	0.81	0.87	0.90	0.90	0.83	1.00	0.93	0.96
Con9-2	0.73	0.01	0.14	0.54	0.02	0.58	0.82	0.74	0.72	0.83	0.80	0.77	0.93	1.00	0.95
Con9-3	0.77	0.02	0.10	0.63	0.01	0.70	0.93	0.89	0.88	0.94	0.93	0.89	0.96	0.95	1.00

Note: Con1, Con3, Con5, Con7, and Con9 represent RCM controls stored at 4 °C for 1, 3, 5, 7, and 9 days, respectively. The suffixes ‘-1’, ‘-2’, and ‘-3’ represent three replicate samples. Con: control; RCM: raw conditioned mutton.

**Table 4:** Spearman correlation of Con5 and preservative-treated samples at the genus level.

	Con5-1	Con5-2	Con5-3	S5-1	S5-2	S5-3	D5-1	D5-2	D5-3	P5-1	P5-2	P5-3	N5-1	N5-2	N5-3
Con5-1	1.00	0.94	0.95	−0.44	−0.02	−0.16	0.69	0.54	0.71	0.92	0.87	0.96	0.85	0.76	0.81
Con5-2	0.94	1.00	0.98	−0.53	−0.12	−0.26	0.52	0.42	0.56	0.87	0.85	0.95	0.87	0.81	0.77
Con5-3	0.95	0.98	1.00	−0.41	−0.01	−0.13	0.59	0.47	0.63	0.89	0.87	0.98	0.89	0.80	0.81
S5-1	−0.44	−0.53	−0.41	1.00	0.62	0.76	−0.16	−0.22	−0.30	−0.55	−0.60	−0.43	−0.57	−0.51	−0.39
S5-2	−0.02	−0.12	−0.01	0.62	1.00	0.55	0.45	0.41	0.18	0.01	−0.03	0.03	0.02	0.12	0.12
S5-3	−0.16	−0.26	−0.13	0.76	0.55	1.00	0.17	0.19	0.11	−0.18	−0.27	−0.10	−0.17	−0.30	−0.06
D5-1	0.69	0.52	0.59	−0.16	0.45	0.17	1.00	0.93	0.85	0.79	0.65	0.71	0.72	0.69	0.69
D5-2	0.54	0.42	0.47	−0.22	0.41	0.19	0.93	1.00	0.79	0.75	0.65	0.62	0.73	0.64	0.67
D5-3	0.71	0.56	0.63	−0.30	0.18	0.11	0.85	0.79	1.00	0.78	0.72	0.75	0.77	0.63	0.82
P5-1	0.92	0.87	0.89	−0.55	0.01	−0.18	0.79	0.75	0.78	1.00	0.96	0.95	0.95	0.79	0.82
P5-2	0.87	0.85	0.87	−0.60	−0.03	−0.27	0.65	0.65	0.72	0.96	1.00	0.92	0.95	0.77	0.81
P5-3	0.96	0.95	0.98	−0.43	0.03	−0.10	0.71	0.62	0.75	0.95	0.92	1.00	0.94	0.82	0.85
N5-1	0.85	0.87	0.89	−0.57	0.02	−0.17	0.72	0.73	0.77	0.95	0.95	0.94	1.00	0.88	0.90
N5-2	0.76	0.81	0.80	−0.51	0.12	−0.30	0.69	0.64	0.63	0.79	0.77	0.82	0.88	1.00	0.82
N5-3	0.81	0.77	0.81	−0.39	0.12	−0.06	0.69	0.67	0.82	0.82	0.81	0.85	0.90	0.82	1.00

Note: Con5 is an RCM control; S5, D5, P5, and N5 denote RCM samples treated with SDA, DHA-S, PLH, and nisin, respectively, all stored at 4 °C for 5 d. Con: control; SDA: sodium diacetate; DHA-S: sodium dehydroacetate; PLH: ε-polylysine hydrochloride; RCM: raw conditioned mutton.

**Table 5:** Results from VITEK 2 gram-negative bacterial identification card (GN) test.

Item	Result	Item	Result	Item	Result	Item	Result	Item	Result
APPA	−	GGT	+	PLE	−	lLATk	−	CMT	+
ADO	−	OFF	+	TyrA	+	AGLU	−	BGUR	−
PyrA	+	BGLU	+	URE	−	SUCT	+	O129R	+
lARL	−	dMAL	−	dSOR	+	NAGA	+	GGAA	+
dCEL	−	dMAN	+	SAC	+	AGAL	+	lMLTa	−
BGAL	+	dMNE	+	dTAG	-	PHOS	−	ELLM	−
H_2_S	−	BXYL	−	dTRE	+	GlyA	-	lLATa	−
BNAG	+	BAlap	−	CIT	+	ODC	+		
AGLTp	−	ProA	+	MNT	−	LDC	+		
dGLU	+	LIP	−	5 KG	+	lHISa	−		

Note: APPA: Ala-Phe-Pro-ARYLAMIDASE; ADO: ADONITOL; PyrA: L-Pyrrolydonyl-ARYLAMIDASE; lARL: L-ARABITOL; dCEL: D-CELLOBIOSE; BGAL: BETA-GALACTOSIDASE; H_2_S: H_2_S PRODUCTION; BNAG: BETA-N-ACETYL-GLUCOSAMINIDASE; AGLTp: Glutamyl Arylamidase pNA; dGLU: D-GLUCOSE; GGT: GAMMA-GLUTAMYL-TRANSFERASE; OFF: FERMENTATION/GLUCOSE; BGLU: BETA-GLUCOSIDASE; dMAL: D-MALTOSE; dMAN: D-MANNITOL; dMNE: D-MANNOSE; BXYL: BETA-XYLOSIDASE; BAlap: BETA-Alanine arylamidase pNA; ProA: L-Proline ARYLAMIDASE; LIP: LIPASE; PLE: PALATINOSE; TyrA: Tyrosine ARYLAMIDASE; URE: UREASE; dSOR: D-SORBITOL; SAC: SACCHAROSE/SUCROSE; dTAG: D-TAGATOSE; dTRE: D-TREHALOSE; CIT: CITRATE(SODIUM); MNT: MALONATE; 5 KG: 5-KETO-D-GLUCONATE; lLATk: L-LACTATE alkalinisation; AGLU: ALPHA-GLUCOSIDASE; SUCT: SUCCINATE alkalinisation; NAGA: Beta-N-ACETYL-GALACTOSAMINIDASE; AGAL: ALPHA-GALACTOSIDASE; PHOS: PHOSPHATASE; GlyA: Glycine ARYLAMIDASE; ODC: ORNITHINE DECARBOXYLASE; LDC: LYSINE DECARBOXYLASE; lHISa: L-HISTIDINE assimilation; CMT: COURMARATE; BGUR: BETA-GLUCORONIDASE; O129R: O/129 RESISTANCE (comp.vibrio.); GGAA: Glu-Gly-Arg-ARYLAMIDASE; lMLTa: L-MALATE assimilation; ELLM: ELLMAN; lLATa: L-LACTATE assimilation. +: positive; −: negative.

**Table 6:** MBC values of PLH and SDA against *Serratia liquefaciens* B2107-1.

	Reagent Concentration (μg/mL)	Colony Growth
PLH	175	-
	350	-
	700	-
	1400	-
SDA	2800	+++
	3200	++
	3600	+
	4000	-

Note: ‘-’ indicates no bacterial growth; ‘+++’, ‘++’, and ‘+’ indicate a large, medium, and small amount, respectively. MBC: minimum bactericidal concentration; PLH: ε-polylysine hydrochloride; SDA: sodium diacetate.

## Data Availability

The original contributions presented in this study are included in the article. Further inquiries can be directed to the corresponding author.

## Abbreviations

The following abbreviations are used in this manuscript:

RCM	Raw conditioned mutton
TVC	Total viable count
TVB-N	Total volatile basic nitrogen
SDA	Sodium diacetate
DHA-S	Sodium dehydroacetate
PLH	ε-polylysine hydrochloride
LAB	Lactic acid bacteria
MIC	Minimum inhibitory concentration
PCA	Plate count agar
OUT	Operational taxonomic unit
LB	Luria–Bertani
GN	Gram-negative bacterial identification card
BA	Biogenic amine
MBC	Minimum bactericidal concentration
PCoA	Principal coordinate analysis
